# Coffee Consumption and Its Potential Implications for Digestive Tract Carcinogenesis

**DOI:** 10.1007/s13668-026-00760-6

**Published:** 2026-04-30

**Authors:** Nevin Sanlier, Ebru Ozler

**Affiliations:** Department of Nutrition and Dietetics, School of Health Sciences, Ankara Medipol University, 06050 Ankara, Altındağ Turkey

**Keywords:** Anticancer effect, Cafestol, Caffeine, Chlorogenic acid, Coffee, Digestive system cancers

## Abstract

**Purpose of Review:**

In this review, the promising benefits of coffee in terms of its composition, anticancer mechanisms, and effects on digestive system cancers are highlighted and suggestions for future research are given.

**Recent Findings:**

Coffee is one of the most widely consumed beverages in the world. Chlorogenic acid, caffeine, cafestol, kahweol, phenolic compounds, alkaloids, trigonelline, and other secondary metabolites in its composition have positive effects on health. Colorectal cancer, esophageal cancer, liver cancer, gastric cancer, and pancreatic cancer are among the most common cancers of the digestive system worldwide. Cancer risk is increasingly influenced by dietary factors.

**Summary:**

Coffee has been linked to potential protective effects against cancer due to its antioxidant, anti-inflammatory, antifibrotic, and antiangiogenic properties, as it contains over 1,000 bioactive compounds. However, there are inconsistent findings concerning coffee consumption and the risk of digestive tract cancers and more clinical studies with larger samples are needed.

## Introduction

Coffee is one of the most widely consumed beverages in the world. It is produced from fruit seeds of plants of the genus *Coffea* (family Rubiaceae), which contains more than ninety species [1]. Together with its stimulating effects and uniquely desirable bitter taste, coffee has many effects on health. These positive effects can be explained by various phytochemicals found in coffee, such as chlorogenic acid (CGA), caffeine, trigonelline, kahweol, and cafestol [2]. These bioactive compounds vary depending on the type of the coffee, its origin, the roasting temperature, extraction procedures, and the degree of grinding. Thanks to the presence of these components and its antioxidant, antimicrobial, antibacterial, and anti-inflammatory properties, coffee may have protective effects against neurodegenerative diseases and certain types of cancer [3]. In addition, it has been reported that coffee consumption may reduce mortality rates associated with heart failure, myocardial infarction, arrhythmia, hypertension, and type 2 diabetes. However, on the other hand, coffee may also have some adverse health effects by increasing blood lipid levels [4]. Some studies in the literature have reported that coffee increases low-density lipoprotein cholesterol (LDL-C), total cholesterol, and triglyceride levels [5, 6]. In recognition of all of these potential effects, coffee has recently become the focus of intense research.

In 2022, there were 9.7 million cancer-related deaths and 20 million new cancer of cases [7]. Colorectal cancer (CRC), esophageal cancer and gastric cancer (GC) are among the most common cancers of the gastrointestinal tract [8]. While 5–10% of digestive system cancers occur as a result of genetic disorders, the remaining 90–95% are caused by adverse environmental conditions and unhealthy lifestyles [9]. Nutrition-related factors are known to affect the risk of cancer, and coffee contains more than 1000 bioactive components with anti-inflammatory, antifibrotic, and antioxidant properties that have the potential to affect carcinogenesis [10]. Many studies have been conducted on coffee intake and the risk of digestive system cancer. Coffee has been reported to exhibit protective properties against certain digestive system cancers, including CRC [11], GC [12], liver cancer [13], and gallbladder cancer (GBC) [14]. However, there are conflicting findings in the literature regarding the relationship between coffee intake and the risk of digestive system cancers.

This study examined the clinical effects and modes of action of coffee, taking into account all of its potential properties and its effectiveness on digestive system cancers while reviewing animal, human, in vivo, and in vitro studies*.*

## Methods

A comprehensive literature review was conducted to identify relevant studies on the relationship between coffee consumption and digestive system cancers. Articles published in English from January 2015 to June 2024 were scanned using the PubMed, Google Scholar, Scopus, and Web of Science databases. The following keywords were utilized while searching those databases: “coffee,” “kahweol,” “cafestol,” “chlorogenic acid,” “caffeine,” “trigonelline,” “decaffeinated coffee,” “green coffee bean,” “roasted coffee,” “oral cavity cancer,” “esophageal cancer,” “liver cancer,” “colorectal cancer,” “gastric cancer,” “pancreatic cancer,” “gallbladder cancer,” and “digestive system cancer.” This time restriction in the selection of articles was applied to ensure the inclusion of the most recent and up-to-date evidence reflecting the current understanding of the relationship between coffee consumption and digestive system cancers.

### Nutritional Composition of Coffee

Beans from the species of *Coffea arabica*, *C. robusta*, *C. liberica*, and *C. excelsa* are among the most commonly used coffee beans [15]. The nutritional composition of coffee beans is complex. A majority of the components of coffee are carbohydrates (60% of the coffee weight). In addition, coffee contains fat, protein, tannins, caffeine, minerals, and other trace elements. The composition of coffee varies depending on the coffee type, origin, and harvest season. The different tastes and colours of coffee beans are created by the chemical reactions of the components of raw coffee beans during the roasting process [16].

Roasting, grinding, and brewing constitute the typical preparation processes for coffee, and during this process, the composition of the coffee undergoes significant changes. Heat-dependent processes that occur during coffee roasting, such as the Maillard reaction and polyphenol oxidation, affect the composition, aroma, and color of the coffee bean. Medium roast is the roasting level at which the formation of bioactive compounds is at its peak [17]. High roasting temperatures cause a decrease in the caffeine and trigonelline concentrations of coffee [18]. Differences in the brewing methods used result in variations in the coffee’s physical properties, caffeine and phenolic compound levels, volatile compound profile, and antioxidant properties [17]. Shorter brewing times allow for the extraction of higher levels of bioactive compounds and lipids [19]. After harvest, coffee is processed using wet, dry, and semi-dry methods, and these methods can significantly affect the coffee’s composition. For example, the wet processing method leads to an increase in free amino acid levels due to protein breakdown. Coffee obtained from the semi-dry processing method has a lower chlorogenic acid concentration and a higher sucrose content compared to coffee obtained from the dry processing method The different types of coffee and processing methods may affect the biological activity of coffee, and consequently its potential health effects [18].

Table 1 presents the nutritional compositions of green coffee beans, brewed coffee, medium and intense roasted coffee, and decaffeinated coffee [20–23].

**Table 1:** Nutritional composition of coffee [20–23]

	Green coffee beans (100 g) (dry)	Medium roast coffee (100 g) (dry)	Intensely roasted coffee (100 g) (drink)	Brewed coffee (100 g) (drink)	Decaffeinated coffee (100 g) (drink)
Energy (kkal)	460	348	33	1	2
Protein (g)	5.29	0	0	0.12	0.12
Fat (g)	15.87	10.9	0	0.02	0.43
Carbohydrate (g)	74	60.9	0	0	0
Fiber (g)	1.9	0	0	0	0
Sugar (g)	58.2	52.2	0	0	0
Sodium (mg)	317	587	47	2	4
Calcium (mg)	271	0	33	2	4
Magnesium (mg)	68	-	-	3	4
Potassium (mg)	1033	-	1170	49	36
Phosphorus (mg)	251	-	-	3	3
Zinc (mg)	0.96	-	-	0.02	0.01
Vitamin C (mg)	0.6	0	-	0	0
Vitamin A (μg)	1	0	-	0	0
Caffeine (mg)	360	117	108	40	1

### Important Compounds found in Coffee in Relation to Cancer Risk

Coffee is known to have chemopreventive properties against various types of cancer. These chemopreventive properties are due to various compounds that utilize different anticancer mechanisms, including caffeine, CGA, and diterpenes such as kahweol and cafestol [24].

**Fig. 1:**
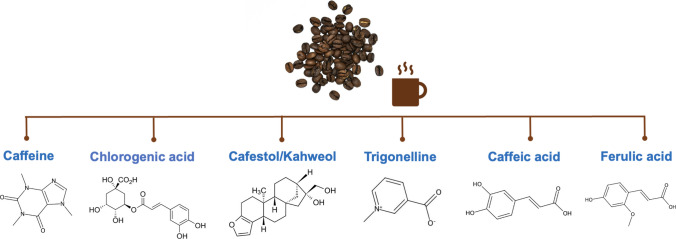
Important compounds found in coffee in terms of cancer risk

Figure 1 illustrates the compounds found in coffee that are important in relation to cancer.

#### Caffeine

Caffeine (1,3,7-trimethylxanthine) is a purine alkaloid belonging to the methylxanthine group [25]. It is found in various plant leaves and seeds and fruit leaves. Coffee beans, tea leaves, cocoa beans, and guarana fruit are among the foods with naturally high caffeine contents [26]. In both humans and animals, caffeine is absorbed in the digestive system after oral intake [25]. The absorption of caffeine is completed within 45 min after oral intake, while blood levels of caffeine reach their peak within 15–120 min. After absorption, caffeine spreads throughout the body before crossing blood–brain barrier. Caffeine is metabolized in the liver by cytochrome P-450 [CYP] enzymes, particularly cytochrome P450 1A2 (CYP1A2). Caffeine metabolites include paraxanthine, theophylline, and theobromine. These metabolites are subsequently metabolized to uric acid and excreted in the urine [27]. After being filtered by the glomeruli, 98% of caffeine is reabsorbed by the renal tubules, with 0.5–2% of unmetabolised caffeine being excreted in urine [25]. Caffeine exerts protective properties against many health problems, including cancer, due to its antioxidant properties, the inhibition of carcinogenesis, various cytoprotective properties, phosphodiesterase inhibition, and its ability to act as a ryanodine and adenosine receptor agonist [28].

#### Chlorogenic Acid

Chlorogenic acid (CGA) is a bioactive polyphenolic compound also known as chlorogenate or 3-CQA, representing a complete quinic acid and ester-hydroxycinnamic group containing coumaroylquinic, dicaffeoyl, feruloyl acids, and caffeoyl. Coffee beans, ferns, tea leaves, cocoa beans, apples, citrus fruits, potatoes, and eggplants are among the foods with high CGA contents [29]. CGA has three groups of chemical compounds: feruloylquinic acids, caffeoylquinic acids, and dicaffeoylquinic acids. The main components of coffee polyphenols are caffeoylquinic acids. The CGA compounds found in coffee contain three chemical compound isomers: neo-chlorogenic acid (3-O-caffeoylquinic acid; 3-CQA), crypto-chlorogenic acid (4-O-caffeoylquinic acid; 4-CQA), and CGA (5-O-caffeoylquinic acid; 5-CQA). The CGA found in green coffee beans includes 3,4-caffeoylquinic acids, 3,5-caffeoylquinic acids, 4,5-caffeoylquinic acids, and 3-, 4-, and 5-caffeoylquinic acids, referred to as total caffeoylquinic acids, and their composition varies depending on the type of coffee. 5-CQA is the most prevalent CGA in coffee beans, accounting for 50% of the total CGA [30]. CGA is hydrolyzed to quinic acid and caffeic acid in the intestine under the action of esterase and subsequently absorbed. After absorption, it is metabolized to sulfate metabolites and glucuronic acid glycosides. The oral absorption of CGA is known to be low. It is present in the plasma as metabolites and is removed from the body via the kidneys [31].

It has been reported that CGA, which has protective effects against many types of cancer, may particularly have protective effects against digestive system cancers such as GC, CRC, and pancreatic cancer (PC) [29, 31]. Matrix metalloproteinase (MMPs) are proteolytic enzymes used by tumor cells during metastasis and are effective in cancer cell growth, differentiation, and apoptosis. CGA inhibits matrix metalloproteinase-9 (MMP-9) in cancer cells. It also affects apoptosis by acting on p53, C-jun nh2-terminal protein kinase (JNK), p21, and nuclear factor erythroid 2-related factor (Nrf2) molecules and regulatory microRNA expression. CGA suppresses cancer progression by modulating signaling via phosphatidyl inositol 3-kinase (PI3K)/protein kinase b (AKT)/phosphatase and tensin homolog (PTEN) pathways. Thus, with all these effects, CGA shows anticancer activity [30].

#### Kahweol and Cafestol

Kahweol is a coffee diterpene molecule found abundantly in Arabica coffee bean oil. On average, there are 661–923 mg of kahweol in 100 g of Arabica coffee [32]. Approximately 70% of kahweol and cafestol are absorbed via the small intestine. Cafestol and its metabolites accumulate to a large extent in the liver and digestive system via the enterohepatic cycle [33]. Kahweol and cafestol exert anticancer effects by inhibiting tumor cell proliferation and tumor metastasis [32, 33].

#### Other Components

Trigonelline is one of the main components found in coffee. It is an alkaloid with antioxidant, anti-inflammatory, and antimicrobial effects. Trigonelline has a high capacity to inhibit Nrf2 transcription [34]. A study reported that using trigonelline-loaded micelles as Nrf2 inhibitors could be an effective way to treat oxaliplatin-resistant CRC [35].

Caffeic acid is a polyphenol belonging to the phenylpropanoid family. Caffeic acid inhibits signal transducer and activator of transcription 3 (STAT3), NFκB, and extracellular signal-regulated kinase 1 and 2 (ERK1/2), activates the nuclear factor erythroid 2-related factor 2/antioxidant response element (Nrf2/ARE) pathway, and exhibits anti-inflammatory, antioxidant, and anticancer properties [36].

Gallic acid (3,4,5-trihydroxybenzoic acid) is a polyphenolic compound that exhibits anti-inflammatory, anticancer, and anti-obesity effects. It can exert anticancer activity by inducing cell cycle arrest, inhibiting metastasis, angiogenesis, and oncogene expression, and promoting apoptosis [37].

Ferulic acid is a phenolic acid compound found in whole grain foods; fruits such as citrus and bananas; vegetables such as beets, cabbage, spinach, and broccoli; and coffee. It possesses antioxidant, antimicrobial, antiviral, antithrombotic, and antitumor properties. Ferulic acid may prevent the proliferation of cancer cells by regulating protein synthesis, modulating the cell cycle, and inducing apoptosis [38].

### Anticancer Mechanisms of Coffee

#### Antioxidant Mechanisms

Abnormal redox homeostasis is observed in cancer cells. Reactive oxygen species (ROS) are protumorigenic and high levels of ROS are cytotoxic. Excessive proliferation of tumor cells causes high ROS production [39]. Many components of coffee show antioxidant activity. Various components stimulate tissue antioxidant gene expression and may provide protection from oxidative stress in the gastrointestinal tract. In addition, they protect cells against ROS and deoxyribose nucleic acid (DNA) strand breakage [40].

#### Anti-inflammatory Mechanisms

Inflammation is frequently associated with the progression and occurrence of cancer [41]. Obesity, autoimmune diseases, bacterial and viral infections, and smoking and alcohol consumption are categorized as external inflammatory factors. In cases of intrinsic inflammation, cancer-induced inflammation is stimulated by cancer-initiating mutations and contributes to malignant progression through the activity of inflammatory cells [42]. Chronic inflammation is characterized by persistent tissue damage, damage-induced cell proliferation, and tissue regeneration. Cell proliferation is associated with metaplasia, a reversible change in cell type. This process is followed by dysplasia, and dysplasia occurs after carcinoma [43]. The phytochemicals and CGA contained in coffee may have protective effects against cancer due to their anti-inflammatory properties [30].

#### Antiangiogenic Mechanisms

Angiogenesis is a process by which blood vessels are formed and it plays a role in both pathological and physiological processes in the body. Tumor angiogenesis is associated with tumor growth, progression, and metastasis [44]. Various biomolecules such as chemokines, growth factors, and adhesion factors are thought to play roles in tumor angiogenesis. In angiogenesis, the expression of proangiogenic factors is increased and antiangiogenic factors are inactivated. All of these factors play a significant role in the pathogenesis of cancer, particularly CRC [45]. Cafestol and kahweol demonstrate protective properties against cancer due to their antiangiogenic effects [40].

#### Antifibrotic Mechanisms

The extracellular matrix components in tumor cells are produced by fibroblasts, the most common type of connective tissue cells. Fibroblasts within tumors are often described as cancer-associated fibroblasts [46]. Activated cancer-associated fibroblasts produce extracellular matrix compounds. The activation and proliferation of cancer-associated fibroblasts occurs in response to soluble signaling molecules secreted by diverse cell types, including blood platelets, immune cells, and cancer cells [47]. It is emphasized that the antifibrotic properties of coffee components may be protective against cancer [40].

#### Apoptosis Induction Mechanisms

Apoptosis is a specific type of programmed cell death that releases phosphatidylserine and also activates proteins of the cysteine-aspartic protease (caspase) family. Other types of programmed cell death used by normal cells and cancer cells include autophagy, pyroptosis, and ferroptosis [48]. Apoptosis is involved in maintaining the balance between cell death and division. Preventing apoptosis causes uncontrolled proliferation of cells and leads to many chronic diseases including cancer [49]. The caffeic acid and CGA found in coffee substantially effect the expression of apoptosis genes and enhance the apoptotic response [24].

#### Protection Against DNA Damage

Coffee may exhibit protective effects against DNA damage through its anti-inflammatory and antioxidant properties. Compounds found in coffee, such as diterpenes and CGA, may protect against DNA damage by reducing inflammation, activating the Nrf2/ARE pathways, lowering ferritin levels, decreasing DNA methylation, and reducing the urinary excretion of oxidative damage markers like 8-hydroxydeoxyguanosine [50]. In one study, chlorogenic acid in coffee was reported to reduce DNA damage and radiation-induced injury in hepatocellular cancers through Nrf2 activation [51].

#### Effects on Autophagy

Autophagy is an intracellular degradation mechanism that involves the formation of double-membraned autophagosomes. It differs from apoptosis by selectively clearing misfolded cytotoxic proteins and inclusion bodies. Autophagy plays a significant role in various pathophysiological processes such as tumor formation, development, cell death, and survival. A complex relationship exists between autophagy and apoptosis in tumor cells. In addition to apoptosis, the PI3K pathway and endoplasmic reticulum stress response potentially play a role in tumour formation mediated by autophagy. Dysregulation in this pathway can affect autophagy levels in tumor cells [52]. A study has reported that caffeine and theophylline induce autophagy and apoptosis in gastric cancer cells through PTEN activation and PI3K/Akt/mTOR pathway suppression, demonstrating anticancer effects [53].

#### Modulation of Transcriptional Factors

CGA found in coffee exerts antioxidant and anti-inflammatory effects by activating the Nrf2/ARE signaling pathway and inhibiting the nuclear factor kappa-B (NF-κB) pathway. It suppresses cell proliferation and angiogenesis through the inhibition of the mitogen-activated protein kinase (MAPK) and PI3K/Akt pathways. Additionally, coffee activates the aryl hydrocarbon receptor (AhR), thereby increasing the expression of cytochrome P450 family 1 subfamily A member 1 (CYP1A1), cytochrome P450 family 1 subfamily A member 2 (CYP1A2), and UDP-glucuronosyltransferases. Through these mechanisms, it supports detoxification and metabolic processes and may exert anticancer effects [50].

### Coffee’s Relationship with Digestive System Cancers

After consumption, the digestive system's organs are the first to be exposed to coffee and its components. Coffee affects the digestive system by stimulating stomach acid production and impacting the production of bile and pancreatic secretions and intestinal motility [54]. At the same time, coffee and its components have anti-inflammatory, antioxidant, and antifibrotic properties and are known to affect carcinogenesis [10].

**Fig. 2:**
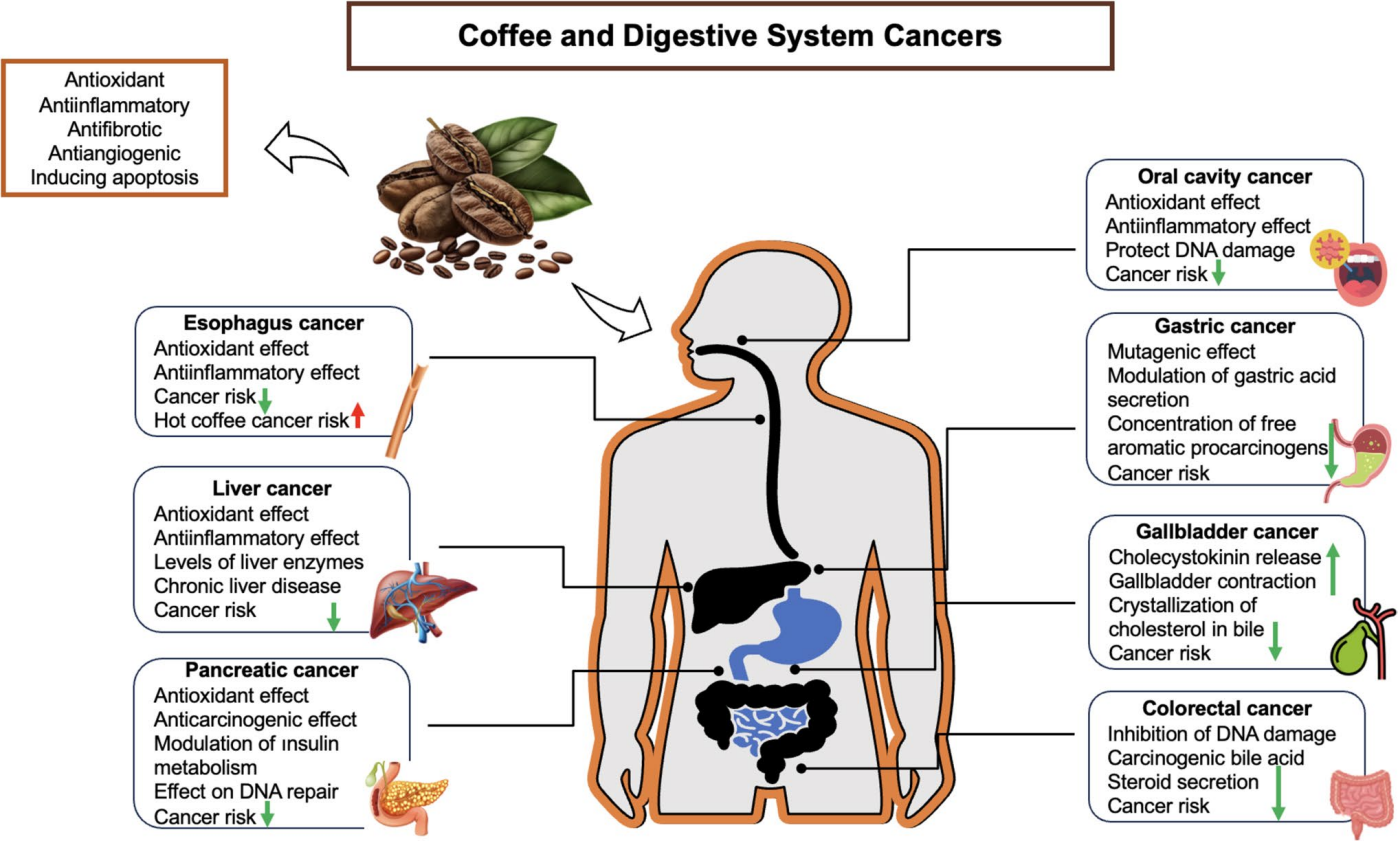
The effect of coffee on gastrointestinal system cancers. **Note:** Arrows indicate the direction of effects (increase or decrease) on both cancer risk and the associated biological processes and mechanisms presented in the figure. Upward arrows (red) indicate increasing effects, whereas downward arrows (green) indicate decreasing effects

Figure 2 shows the effect of coffee on digestive system cancers.

#### Oral Cavity Cancer

Oral cavity cancer encompasses cancers of the mouth, lips, and/or tongue. Tobacco use and alcohol are considered to be major risk factors. Lymphomas, salivary gland malignancies, sarcomas, and mucosal melanomas may all occur in the oral cavity. However, oral squamous cell carcinoma (OSCC) is the most common cancer of the oral cavity [55]. In 2020, on a global level, 378,000 new diagnoses and 178,000 deaths due to oral cavity cancers were recorded [56]. While 188,230 individuals died of lip and oral cavity cancers in 2022, 389,485 individuals were newly diagnosed [7]. The interplay between coffee intake and cancer has been investigated for many years. It is thought that coffee may protect against oral cancer due to its antioxidant compounds [57].

He et al. [57] stated that moderate and high levels of coffee intake may be protective against oral cavity cancer. However, another meta-analysis study found that there was no relationship between high coffee intake (≥ 6 cups/day) and oral cancer [58]. In a systematic review evaluating the effects of coffee components on OSCC, it was noted that the quercetin and epigallocatechin gallate found in coffee might have anticancer effects. The same review highlighted a dose-dependent relationship between these components and OSCC [59]. However, the literature lacks sufficient evidence on the therapeutic effects of coffee components in cases of oral cancer. The existing evidence is largely observational, and meta-analyses report inconsistent results. This inconsistency may stem from differences in the included studies and exposure categorization, which limits the generalizability of the findings.

#### Esophageal Cancer

Esophageal cancer takes different forms including esophageal squamous cell carcinoma (ESCC), esophageal adenocarcinoma (EAC), small-cell carcinomas, lymphomas, melanomas, and sarcomas [60]. The most common types are ESCC and EAC [61]. EAC begins in gland cells located mainly in the lower thoracic esophagus [60]. The condition known as Barrett’s esophagus is a complicated form of gastroesophageal reflux disease characterized by mucosal dysplasia and which can result in EAC [62]. ESCC originates in the squamous cells that line the oesophagus and primarily affects the cervical or upper thoracic oesophagus. [60]. Esophageal cancer has been reported to cause more than 500,000 new diagnoses and nearly 500,000 deaths each year. It is the eleventh most common type of cancer and the seventh leading cause of cancer-related deaths [7]. Age, sex, genetics, race, socioeconomic status, obesity, nutrition, and smoking and alcohol use are among the risk factors [61]. Coffee irritates the esophageal mucosa when consumed at high temperatures. This can lead to the endogenous formation of reactive nitrogen species followed by nitrosamines, which are strong carcinogens [63].

A recent study reported that there is no association between coffee consumption and oesophageal cancer [63]. This result may be related to the use of Mendelian randomisation, a method that reduces the effects of confounding factors and reverse causality. Another study reported that coffee consumption had a protective effect against esophageal cancer in East Asians, but no such effect was observed in Americans of European origin [64]. This difference in the study findings may be explained by population-specific factors such as coffee consumption habits, genetic factors, and lifestyle behaviors. Carter et al. [10] stated that coffee intake is associated with esophageal cancer, and the temperature factor may be particularly effective in this relationship. The association observed in the study may be related more to the temperature factor than to the composition of coffee. High-temperature coffee consumption may lead to oesophageal damage and carcinogenesis. It has also been stated that CGA, a component of coffee, reduces the movement of cancer cells via the epidermal growth factor receptor (EGFR)/phosphorylated protein kinase b (p-AKT)/Snail pathway and may exert an antimetastatic effect against ESCC [65]. Another study reported that CGA suppressed the progression of ESCC and downregulated BMI1 and SOX2 expression; thus, it may be said to have antitumor effects [66]. The observed heterogeneity across studies may be attributed to differences in the study populations (e.g. ethnicity), the temperature at which the drinks were consumed, and the methodological approaches adopted. This limits the ability to establish a causal relationship and makes generalization difficult.

#### Gastric Cancer

Gastric cancer (GC) is a multifactorial illness as many different factors, both genetic and environmental, play a role in its development [67]. It is the fifth most common type of cancer and the fourth leading cause of cancer-related deaths worldwide [68]. Conventional GC, hereditary diffuse GC, early-onset GC, and gastric stump cancer are among the subtypes of GC. The most common type of GC is tubular adenocarcinoma, which can be classified into papillary and mucinous types, as well as cardia and non-cardia depending on the anatomical location [54]. Signet ring cell carcinoma accounts for roughly 10% of GC cases, and more than 50% of these tumors are defined by the appearance of signet ring cells. Smoking and alcohol consumption, nutrition, family history, and Epstein-Barr virus and *Helicobacter pylori* infections are among the risk factors for GC [67]. Nutrition is seen as a particularly important risk factor. Salt-preserved foods, large amounts of processed meat, grilled or barbecued meat consumption, and low levels of fruit consumption increase the risk of GC [7]. There are many mutagenic and genotoxic components in the composition of coffee. Caffeine can affect DNA repair and cell apoptosis. Phenolic compounds found in coffee are thought to have protective effects against cancer, and certain components may prevent mutagenic effects by reducing the concentration of free aromatic procarcinogens required for cytochrome activation [69].

A recent study reported that there was no important relationship between coffee intake and GC; only a positive relationship between gastric cardia cancer and coffee intake [five or more cups per day] was found [70]. In this study, coffee consumption was strongly associated with gastric cardia cancers. This indicates that coffee consumption has a region-specific effect on the stomach. An ecological study identified an inverse linear correlation between annual coffee consumption in kilograms per capita and the estimated age-adjusted incidence and mortality rates of GC across 25 countries [71]. The results of this study point to the effects of coffee at the population level rather than individual consumption. Also, since this is a population-level assessment, it should be considered that many confounding factors may be at play. Kim et al. [12] found that the risk of GC was lower in individuals who consumed > 60 cups of coffee per month. Fakhri et al. [72] reported that coffee intake decreased the risk of GC. This meta-analysis may have been affected by heterogeneity among the included studies (case–control, cohort, and cross-sectional) and differences in the assessment of coffee exposure. Other studies have stated that there is no relationship between coffee consumption and GC [63, 69, 73]. The findings regarding the relationship between coffee consumption and GC are inconclusive. While some studies report a protective effect, others do not show a significant association. These results may vary depending on the amount of consumption, anatomical tumor localization, and the methodologies used in the studies.

#### Gallbladder Cancer

Gallbladder cancer [GBC] is a hepatobiliary malignancy that occurs on the mucosal surface of the gallbladder [74]. It is asymptomatic in the early period, has a high mortality rate, and is among the most common cancers of the biliary tract [7, 74, 75]. Some beverages, sugar, body mass index, chronic diseases [particularly diabetes], age, race, infections, and gallbladder stones are among the risk factors [74]. Although the causes of GBC are not yet fully known, gallstones have been identified as a risk factor [76]. It is thought that the nutritional factors that cause gallstone formation may also cause GBC [77]. Coffee may stimulate the secretion of cholecystokinin, enhance gallbladder contraction, and reduce cholesterol crystallization in bile [76, 77].

In the study by Huang et al. [78], it was found that coffee consumption was positively associated with the incidence of GBC compared to non-coffee drinkers. However, a clear dose–response relationship was not observed among coffee drinkers. This may be due to the categorization of coffee consumption amounts. In another study, high coffee consumption was significantly associated with a lower risk of GBC [14]. This result was observed in a population already matched for gallstones, which is a high-risk group. However, a previous meta-analysis reported no relationship between coffee consumption and biliary tract cancer [77]. In a prospective cohort study conducted in Sweden, consuming one additional cup of coffee every day was found to reduce the risk of GBC by 14% [76], while Tran et al. [79] stated that consumption of decaffeinated coffee increased the risk of GBC. However, in the latter study, it was also stated that there was no dose–response association between coffee and GBC and that the effect would be meaningless with further analysis. This inconsistency between studies may stem from differences in the types of coffee consumed (e.g., decaffeinated coffee). A case–control study carried out in India revealed a statistically significant inverse relationship between coffee consumption and the risk of developing GBC [80]. However, the sample group for this study is relatively small, and the relationship between many diet-related factors and gallbladder cancer was evaluated; therefore, careful interpretation of the results is advisable. Studies evaluating the relationship between coffee consumption and GBC have yielded conflicting results. The findings may vary according to the amount and type of coffee consumed, as well as the population in which the study was conducted.

#### Liver Cancer

Primary liver cancer is divided into two groups: hepatocellular carcinoma (HCC) and intrahepatic cholangiocarcinoma [81]. Accordingly, 80–90% of primary liver cancers are HCC and 10–15% are cholangiocarcinoma [82]. Liver cancer causes nearly 800,000 deaths each year. It is the sixth most common type of cancer and the third leading cause of cancer-related deaths [7]. Smoking, diabetes, hepatitis B and C viruses, alcohol-related cirrhosis, fatty liver disease, and various dietary exposures are among the risk factors for liver cancer. The liver is involved in carbohydrate, fat, and protein metabolism as well as detoxification and hormone production. Therefore, nutrition is an important factor in liver cancer [83]. Coffee may lower the levels of liver enzymes and decrease the risk of chronic liver illness. Thus, it is thought that coffee may be protective against liver cancers, especially HCC [84].

Studies have found inverse relationships between coffee intake and liver cancer or HCC [13, 84–87]. However, while the Mendelian randomization study, which found an inverse relationship, presented a stronger result due to the reduction of confounding factors, the meta-analysis studies may have been affected by some confounding factors. Therefore, it is beneficial to interpret the results carefully. A meta-analysis study showed that high coffee consumption significantly diminished the risk of liver cancer [88]. The results of this study are strong because of the inclusion of prospective cohort studies with low heterogeneity. However, the inclusion of only a specific population limits its generalizability. Bhurwal et al. [89] associated higher coffee consumption with an important decrease in the likelihood of developing liver cancer or specifically HCC. It has been found that the incidence of liver cancer is lower in those who consume > 60 cups of coffee per month [12]. However, this study has a cross-sectional design, and causal interpretation is limited; therefore, the results should be interpreted carefully. Another cohort study showed an inverse relationship between ground and instant coffee intake and HCC [79]. The study had a large sample size and evaluated different types of coffee. However, the assessment of coffee consumption based on self-reported intake may have influenced the study results. Yu et al. [90] reported that coffee consumption reduced the risk of HCC, and the low heterogeneity among the included studies strengthens the reliability of this finding. In another study, CGA, a component of coffee, was reported to have the potential to suppress human hepatoma cells and may be effective in terms of anticancer mechanisms [91]. This finding is based on in vitro data and may not directly reflect population-level effects. Chen et al. [92] reported that coffee consumption was not associated with HCC. The inclusion of individuals with chronic hepatitis B infection in this study may have influenced the interpretation of the effect of coffee on cancer through virus-related carcinogenic mechanisms. Although the majority of studies investigating the relationship between coffee consumption and liver cancer suggest a protective effect, some studies have found no significant association. This situation may be explained by factors such as differences in study design (e.g., cross-sectional vs. cohort), underlying conditions such as chronic hepatitis infection, and the reliance on self-reported coffee consumption.

#### Pancreatic Cancer

Pancreatic cancer (PC) occurs when abnormal DNA mutations in the pancreas cause cells to grow and divide in an uncontrolled way and form tumors [93]. Age, sex, race, genetics, smoking and alcohol consumption, obesity, diabetes, nutrition, the microbiota, infection, and pancreatitis are among the risk factors [93, 94]. PC is the common term used for a malignant tumor called adenocarcinoma that occurs in the epithelial cells of the glandular structures in the ductal cells of the pancreas. Pancreatic ductal adenocarcinoma constitutes the majority of PC [93].

In a prospective cohort study including individuals who did not smoke, no relationship was found between coffee consumption and PC [95]. The exclusion of smoking as a significant confounding factor is one of the strengths of this study. Similarly, another study also found no significant relationship between coffee consumption and PC [96]. Li et al. [97] reported that a daily increase in coffee consumption by one cup increased the risk of PC by 5.87%. The effect size appears to be relatively small in this study, therefore careful interpretation of the results is advisable. In patients with PC, it was found that a diet aimed at reducing the risk of diabetes reflected no relationship between coffee consumption and PC [98]. In this study, the effect of coffee on pancreatic cancer was evaluated within a composite dietary score, and its individual effect may have been diluted by the stronger influence of other dietary components. It has also been determined that CGA, a component of coffee, inhibits the growth of PC cells through AKT/glycogen synthase kinase-3 beta (GSK-3β)/β-catenin signaling [99]. In another study, it was reported that CGA suppressed pancreatic ductal adenocarcinoma cell growth both in vitro and in vivo [100]. Although in vivo and in vitro experimental studies using CGA have yielded positive results, these effects may not directly translate to population-level outcomes due to differences in dose and bioavailability. There is no strong or consistent epidemiological evidence to suggest that coffee consumption is linked to pancreatic cancer. Although some studies have reported modest increases in risk, most have found no significant association. On the other hand, some experimental findings suggest that coffee components such as chlorogenic acid may have inhibitory effects on the growth of pancreatic tumor cells.

#### Colorectal Cancer

Colorectal cancer (CRC) is the third most common type of cancer and the second biggest cause of cancer-related deaths [7]. By 2035, there will be an estimated 2.5 million new cases worldwide [101]. High-fat diets, high levels of red meat consumption, insufficient consumption of milk and dairy products or fruits and vegetables, and low intake of fiber, calcium, and vitamin D are among the nutrition-related risk factors of CRC [102]. Several mechanisms have been proposed to explain coffee's ability to protect against CRC. These include increasing colon motility in the rectosigmoid region and reducing carcinogenic bile acid and sterol secretion in the intestine. In addition, coffee contains bioactive compounds such as polyphenols and caffeine, which possess antitumorigenic and antioxidant properties [103].

In a meta-analysis study, no important relationships between coffee consumption and colon cancer were identified [104]. This study only considered Asian populations, therefore the generalizability of the findings is limited. Kim et al. [12] reported that the risk of colon cancer in individuals who consume more than 60 cups of coffee per month is higher compared to those who do not drink coffee. In this study, coffee consumption was determined based on individuals' self-reported information, and it is a cross-sectional study; therefore, limited causal inferences may be possible. In another study, high coffee intake (≥ 3 cups per day) was associated with a reduced likelihood of developing CRC. Consumption of coffee additives such as cream and sugar strengthened this inverse relationship [11]. The presence of coffee additives, such as cream and sugar, may act as important confounding factors that influence the observed association. In another meta-analysis study, it was stated that the relationships between coffee intake and CRC risk differed across different populations and the results were controversial [105]. Um et al. [106] found that consuming ≥ 2 cups of decaffeinated coffee per day was associated with a reduced risk of colorectal, rectal, and colon cancer compared to not consuming decaffeinated coffee. In this study, coffee consumption was assessed using self-reported dietary frequency questionnaires, and smoking status may have been a significant confounding factor affecting the results. In one study, coffee and green tea consumption were not linked to an increased risk of CRC; however, an increased risk of colon cancer was reported among women who consumed black coffee [103]. This finding may be affected by the type of coffee consumed. A cell study reported that the CGA-rich fraction obtained from green coffee bean extract can be used as an alternative treatment together with CRC surgical treatment to increase the therapeutic efficiency and survival rate [107]. The findings of this study are based on in vitro data; therefore, their clinical relevance may be limited. Lu et al. [108] stated that consuming 3 or more cups of coffee per day could reduce the risk of CRC by 77% in general and by 83% in men. However, the findings of this study may not be applicable to other populations due to genetic interactions. Furthermore, the assessment of coffee consumption based on self-reporting may be subject to recall bias. A systematic review reported that there is insufficient proof that caffeinated coffee reduces the risk of CRC and that the protective effect of drinking coffee against CRC is only evident with the consumption of > 5 cups/day [109]. A study conducted on rats indicated that coffee may have chemoprotective effects against CRC [110]. Because these results were obtained from animal studies, their generalizability to humans is limited. Another study reported that coffee intake had no effect on the incidence of CRC subtypes based on T-cell classification [111]. Rosato et al. [112] reported no meaningful link between coffee consumption and CRC risk; the overall odds ratio for total coffee intake was 0.99, and no significant trend was observed with respect to consumption dose or duration. The case–control design of this study may limit the interpretation of the findings. In another study investigating coffee and alcohol intake and CRC risk, an inverse relationship between coffee intake and CRC risk was found [113]. In this study, Mendelian randomization was used, which may limit the generalizability of the results. It has been reported that coffee consumption in patients with CRC does not significantly differ with respect to cancer-specific mortality, lymphocytic reactions, or levels of any T-cell subsets [114]. Another study reported that drinking more than four cups of coffee per day was associated with a 32% reduced risk of colorectal recurrence compared to drinking fewer than two cups per day. Additionally, the study described a U-shaped relationship between coffee drinking and all-cause deaths, with the lowest risk observed among people who drank 4 cups of coffee per day [115]. In this study, as in some other studies, coffee consumption is based on self-report by individuals. In another meta-analysis study, it was stated that an increase of 150 mL in daily coffee intake could reduce the risk of colon adenoma [116]. Mackintosh et al. [117] reported that in patients with advanced or metastatic CRC, drinking an extra cup of coffee each day was linked to a lower risk of disease progression and death. The observational design of the study and the assessment of coffee consumption based on self-reported data may limit the strength of the evidence. Studies in the literature examining the relationship between coffee drinking and colorectal cancer have shown conflicting results. While some studies have reported protective effects, others have found no significant association, and a few have indicated an increased risk in specific groups. These differences may be attributed to factors such as the reliance on self-reported coffee consumption and variations in study populations and study designs (e.g., case–control, cross-sectional, and observational cohort studies).

In vivo, in vitro, animal, and human studies conducted to assess the impact of coffee on gastrointestinal tract cancers are summarized in Table 2.

**Table 2:** Studies on Coffee consumption and gastrointestinal cancers

Cancer type	Cancer risk		Sample/Intervention	Outcomes	Ref
Oral squamous cell carcinoma	±		Case group: OSCC patients (n = 593) Control group: Healthy (n = 1228)	No significant association was observed between coffee intake and OSCC	[118]
Oral squamous cell carcinoma	↓		Different CGA concentrations ranging from 500–2500 µM were applied to the OSCC cell line	Expression of p21 and p53 was downregulated after CGA treatment. CGA showed antiproliferative and cytotoxic effect on OSCC cell line	[119]
Esophageal cancer	↑		Case group: Esophageal cancer patient (n = 104) Control group: Healthy (n = 208)	High coffee drinking and very hot coffee consumption increased the risk of esophageal cancer	[120]
Gastric cancer	↓		Meta-analysis study (9 cohort and 13 case–control studies) 7.631 cases 1.019.693 controls	It has been suggested that coffee consumption may be associated with a reduced risk of GC	[121]
Gastric cancer	↑		Meta-analysis study (13 cohort studies) 1.324.559 individuals 3.484 cases of GC	It has been reported that there is a significantly increased risk between gastric cardia cancer and coffee consumption (RR: 1.50). It has been stated that high amounts of coffee consumption is a risk factor for GC	[122]
Gastric cancer	± ↑		477.312 individuals, 11.6 years of follow-up, 683 diagnoses of gastric adenocarcinoma	No association was found between total coffee, caffeinated and decaffeinated coffee consumption and the overall risk of GC. However, total and caffeinated coffee consumption has been associated with an increased risk of gastric cardia cancer	[123]
Gallbladder carcinoma	↓		Bile duct cancer cases (n = 627)	Coffee consumption reduced the risk of gallbladder and extrahepatic bile duct cancer in women	[124]
Bile duct cancer	±		1.138.623 individuals aged 45–74, 15-year follow-up, 284 biliary tract cancer diagnoses	Coffee has been found to have no clear association with bile duct, gallbladder, and extrahepatic bile duct cancer	[125]
Hepatocellular carcinoma	↓		Phospholipid-based in situ gel containing CGA was applied in vitro or in *vivo*	CGA suppressed tumor growth without any side effects	[126]
Hepatocellular carcinoma	↓		Human HCC HepG2 cells were treated with CGA (0, 250, 500, and 1000 μM for 48 h). Proliferation of HCC cells in vivo has been detected. The expression of DNMT1, p53, p21, p-ERK, MMP-2 and MMP-9 in tumors was investigated	CGA inhibited the proliferation and metastasis of HepG2 cells both in vitro and in vivo by downregulating DNMT1 protein expression, which increased p53 and p21 activity. It has been reported that CGA can be used as chemopreventive for HCC	[127]
Hepatocellular carcinoma	↓		215.000 individuals, 18 years of follow-up, 451 HCC diagnoses	Compared to non-coffee drinkers, a 38% decrease in the risk of HCC was found in those who drank 2–3 cups of coffee per day, and a 41% decrease in the risk of HCC in those who drank more than 4 cups of coffee per day	[128]
Hepatocellular carcinoma	↓		486.799 individuals, 11 years of follow-up, 201 HCC diagnoses	It has been reported that the risk of HCC is 72% lower in coffee consumers in the lowest quintile compared to coffee consumers in the highest quintile, and that this relationship is valid for caffeinated coffee, while there is no relationship between decaffeinated coffee and cancer risk	[129]
Liver cancer	↓		167.720 individuals, average follow-up of 15.3 years, 34,031 cancer diagnoses	It has been reported that coffee intake (≥ 4 cups/day) may reduce the risk of liver cancer. (HR: 0.57)	[130]
Liver cancer	↓	Group 1: Liver cancer (n = 4221) Group 2: Fatal liver disease (n = 4242) The effect of coffee consumption and coffee metabolites on liver cancer and fatal liver disease was investigated (27 years of follow-up)		Metabolites have been identified that are strongly associated with coffee consumption, liver cancer, and liver disease mortality. Tyrosine, trigonelline, glucocenodeoxycholic acid, and glycocholic acid have been reported to be inversely associated with both diseases	[131]
Pancreatic cancer	↓	193.439 individuals Heavy filtered coffee consumption (≥ 4 cups/day) Light filtered coffee consumption (≤ 1 cup/day)		It has been reported that increased consumption of filtered coffee may reduce the risk of PC (HR: 0.74)	[132]
Pancreatic cancer	±	457.366 individuals, 1541 diagnosed with primary PC		It was found that there was no relationship between total coffee, caffeinated and decaffeinated coffee intake and PC	[133]
Colorectal cancer	↓	Male webster mice (1,2-dimethylhydrazine/deoxycholic acid-induced colon carcinogenesis) intragastrically five times a week for ten weeks Group 1: CAF (50 mg/kg) (n = 10) Group 2: CGA (25 mg/kg) (n = 10) Group 3: CAF + CGA (50 + 25 mg/kg) (n = 10)		CAF + CGA reduced epithelial cell proliferation and increased apoptosis in colonic crypts. It reduced IL-6, IL-17 and TNF-α levels. OncomiR in the colon downregulated miR-21a-5p. It has been reported that combination therapy reduces early-stage colon carcinogenesis and may prevent CRC	[134]
Colorectal cancer	↓	Stage 1, 2 and 3 CRC cases (n = 1599), mean follow-up of 7.8 years There were 803 deaths, 188 of which were due to CRC		Individuals who consumed at least 4 cups of coffee per day had a 52% reduced risk of CRC-specific death compared to nondrinkers (HR: 0.48). High intake of caffeinated and decaffeinated coffee (≥ 2 cups/day) reduced the risk of CRC-specific mortality and all-cause mortality	[135]
Colorectal cancer	↓	Kahweol was dissolved in dimethyl sulfoxide and applied to human CRC cells		Kahweol induced apoptosis via ATF3 in human CRC cells	[136]
Colorectal cancer	↓	Case group: CRC patient (n = 5145) Control group: Healthy individual (n = 4097)		Coffee consumption was associated with a 26% lower risk of CRC (decaffeinated coffee). Coffee consumption was found to be inversely associated with CRC risk in terms of dose–response	[137]
Colorectal cancer	↑	83.778 postmenopausal women, 12.9 years of follow-up, 1282 CRC diagnoses		It has been determined that moderate and high levels of coffee consumption increase the risk of CRC. Moderate consumption of distilled coffee and high intake of distilled coffee have been associated with increased CRC risk	[138]
Colorectal, liver and esophageal cancer	↓↓↑	922.896 individuals aged 28–94, 30 years of follow-up, 118.738 deaths due to cancer		Among nonsmokers, an increase in coffee consumption of 2 cups per day was found to be inversely associated with death from CRC (HR: 0.97) and liver (HR: 0.92) cancers, and positively associated with death from esophageal cancer (HR: 1.07)	[139]

↑ increased risk; ↓ decreased risk; +/− no or inconsistent association; multiple symbols indicate differing results across cancer types, subgroups, or analyses

CAF: caffeine; CGA: chlorogenic acid; CRC: colorectal cancer; DNMT1: DNA methyltransferase 1; GC: gastric cancer; HCC: hepatocellular carcinoma; IL-6: interleukin 6; IL-17: interleukin 17; MMP-2: matrix metalloproteinase-2; MMP-9: matrix metalloproteinase-9; OSCC: oral squamous cell carcinoma; PC: pancreatic cancer; TNF-a: tumor necrosis factor-a

### Safe Intake Level and Toxic Effects

The safe intake level for caffeine in coffee has been determined as 200 mg or almost 3 mg/kg [140] and 100 g of brewed coffee contains 40 mg of caffeine [21]. The US Dietary Guidelines Advisory Committee has stated that consumption of up to 400 mg of caffeine per day, or 3–5 cups of coffee, is not linked to an increased risk of cancer [141]. Furthermore, the European Food Safety Authority (EFSA) has stated that daily consumption of up to up to 400 mg of caffeine does not raise safety issues for non-pregnant adults. For pregnant women, a daily intake of up to 200 mg is considered safe and does not pose a risk to the fetus. Similarly, breastfeeding women can safely take up to 200 mg of caffeine per day without safety concerns for the breastfed infants [140].

## Conclusion and Recommendations

The CGA, caffeine, kahweol, cafestol, and phenolic components found in coffee exert antioxidant, antifibrotic, anti-inflammatory, and antiangiogenic effects. It is thought that coffee may be protective against cancer as a result of suppressing ROS, modulating inflammation, and inhibiting the angiogenesis of tumor cells. Coffee also has effects on the digestive system by stimulating stomach acid production, affecting the production of bile and pancreatic secretions, and impacting intestinal motility. These effects suggest that coffee may be protective against digestive system cancers, but studies on this subject are inconsistent. Therefore, it is not yet possible to make generalizations about the effects of coffee on markers of digestive system cancers. More in vivo, in vitro, cell, animal, human, and epidemiological studies on this subject are needed in clinical settings. Appropriate evidence and more detailed research are required to confirm the beneficial effects of coffee in the prevention, treatment, and management of the digestive system cancers, as discussed in this review.

### Future Perspective

As the studies reviewed here show, the use of coffee in preventing and treating digestive system cancers is a popular and rapidly growing topic of debate. Although there is considerable interest in the potential beneficial effects of coffee in the prevention and treatment of cancers of the digestive system, clear evidence of the protective effects of coffee against cancer is still lacking. There are still conflicting data on some aspects of the effects of coffee on digestive system cancers and it is necessary to clarify and strengthen the existing evidence. In this context, this review will serve as a guide for evaluating the efficacy and safety of coffee and assessing its pharmacological and clinical effects, its mechanisms of impact, and the risks related with high coffee intake through animal, human, in vivo and in vitro studies.

Future studies should address the key gaps in the literature on this topic. Further mechanistic research is required to improve our understanding of the biological processes through which coffee and its bioactive components influence digestive system cancers. To improve comparability between studies, coffee consumption should be standardised, taking into account different types, doses and preparation methods. Furthermore, more randomised controlled and prospective cohort studies are required to elucidate causal relationships more clearly. Future studies should also control for confounding factors such as alcohol use, smoking status, physical activity, and dietary habits.

## Key References

- Liu X, Yu H, Yan G, Xu B, Sun M, Feng M. Causal relationships between coffee intake, apolipoprotein B and gastric, colorectal, and esophageal cancers: univariable and multivariable Mendelian randomization. Eur J Nutr. 2024;63:469–83. 10.1007/s00394-023–03281-y.
  - This study investigated the potential causal relationship between coffee consumption and gastrointestinal cancers using the Mendelian randomization method. No causal association was observed between coffee consumption and gastric, colorectal, or esophageal cancers.
- Chen X, Liu B, Tong J, Bo J, Feng M, Yin L, Lin X. Chlorogenic acid inhibits proliferation, migration and invasion of pancreatic cancer cells via AKT/GSK-3β/β-catenin signaling pathway. Recent Pat Anticancer Drug Discov. 2024;19:146–53. 10.2174/1574892818666230327134746.
  - This study reported that chlorogenic acid inhibits proliferation, migration, and invasion in pancreatic cancer cells by suppressing the AKT/GSK-3β/β-catenin signaling pathway. The findings provide important mechanistic evidence that chlorogenic acid may exert antitumor effects through this pathway.
- Oyelere AM, Kok DE, Bos D, Gunter MJ, Ferrari P, Keski-Rahkonen P, et al. Coffee consumption is associated with a reduced risk of colorectal cancer recurrence and all-cause mortality. Int J Cancer. 2024;154:2054–63. 10.1002/ijc.34879.
  - This study is a prospective cohort study investigating the association between coffee consumption and the risk of disease recurrence and all-cause mortality in individuals with colorectal cancer. Drinking more than four cups of coffee per day was reported to reduce the risk of colorectal cancer recurrence compared to drinking fewer than two cups per day.

## Data Availability

No datasets were generated or analysed during the current study.

## Abbreviations

3-CQA: 3-O-caffeoylquinic acid
4-CQA: 4-O-caffeoylquinic acid
5-CQA: 5-O-caffeoylquinic acid
AhR: Aryl hydrocarbon receptor
AKT: Protein kinase b
CGA: Chlorogenic acid
CRC: Colorectal cancer
CYP1A1: Cytochrome P450 family 1 subfamily A member 1
CYP1A2: Cytochrome P450 family 1 subfamily A member 2
DNA: Deoxyribose nucleic acid
EAC: Esophageal adenocarcinoma
EFSA: European Food Safety Authority
EGFR: Epidermal growth factor receptor
ERK 1/2: Extracellular signal-regulated kinase 1 and 2
ESCC: Esophageal squamous cell carcinoma
GBC: Gallbladder cancer
GC: Gastric cancer
GSK-3β: Glycogen synthase kinase-3 beta
HCC: Hepatocellular carcinoma
JNK: C-jun nh2-terminal protein kinase
LDL-C: Low-density lipoprotein cholesterol
MAPK: Mitogen-activated protein kinase
MMP-9: Matrix metalloproteinase-9
MMP: Matrix metalloproteinase
NF-κB: Nuclear factor kappa-B
Nrf2: Nuclear factor erythroid 2-related factor
Nrf2/ARE: Nuclear factor erythroid 2-related factor 2/ Antioxidant response element
OSCC: Oral squamous cell carcinoma
p-AKT: Phosphorylated protein kinase b
PC: Pancreatic cancer
PI3K: Phosphatidyl inositol 3-kinase
PTEN: Phosphatase and tensin homolog
ROS: Reactive oxygen species
STAT3: Signal transducer and activator of transcription 3
